# Antimicrobial Resistance in Major Gram-Positive Pathogens: Beyond the Vancomycin Border

**DOI:** 10.3390/antibiotics15080780

**Published:** 2026-08-13

**Authors:** Despoina Papageorgiou, Karolina Akinosoglou

**Affiliations:** 1 Faculty of Medicine, University of Patras, Rio, 26504 Patras, Greece; dspn.pap96@gmail.com; 2 Department of Internal Medicine, University General Hospital of Patras, Rio, 26504 Patras, Greece; 3 Division of Infectious Diseases, University General Hospital of Patras, Rio, 26504 Patras, Greece

**Keywords:** multidrug-resistant, Gram-positive, linezolid, daptomycin, dalbavancin, ceftaroline, combination therapy

## Abstract

Antimicrobial resistance (AMR) remains an ongoing and critical concern with substantial consequences for global public health. Although recent attention has focused on managing Gram-negative infections, Gram-positive pathogens contribute comparably to morbidity and mortality. The increasing prevalence of multidrug-resistant (MDR) Gram-positive infections has led to the widespread use of vancomycin and other next-line agents including linezolid, daptomycin, ceftaroline and dalbavancin. However, emerging resistance to these agents is increasingly reported, further limiting the available therapeutic options for Gram-positive infections. To this end, there is an urgent need for effective strategies to overcome resistance, including combination therapy, optimization of pharmacokinetic and pharmacodynamic parameters, and improved source control to reduce infection burden. Additionally, resistance surveillance, antimicrobial stewardship and the development of novel antimicrobials are warranted to address the rising threat of resistant Gram-positive infections. This review aims to provide an overview of resistance mechanisms in major Gram-positive pathogens and discuss existing and emerging approaches to overcome resistance to agents beyond vancomycin, incorporating recent evidence on combination therapies and providing a practical treatment algorithm.

## 1. Introduction

Antimicrobial resistance (AMR) remains an ongoing and critical concern with substantial consequences for global public health. Recent estimates have shown that 4.71 million deaths were associated with bacterial AMR in 2021, including 1.14 million directly attributable deaths, a burden that could rise to 1.91 million attributable deaths and 8.22 million deaths associated with AMR by 2050 [1]. Although much of the recent attention has focused on managing Gram-negative infections—which, despite advancements in antimicrobial research, remain among the WHO critical priority pathogens due to their diverse resistance mechanisms—the impact of Gram-positive pathogens on morbidity and mortality is substantial in both community and healthcare settings [2]. Methicillin-resistant *Staphylococcus aureus* (MRSA) and vancomycin-resistant *Enterococcus faecium* are still listed as high-priority pathogens according to the WHO list, along with other notable additions including macrolide-resistant Group A streptococci, penicillin-resistant Group B streptococci*,* and macrolide-resistant *S. pneumoniae* which can pose a significant threat especially among high-risk populations [2,3]. The prevalence of resistance in Gram-positive bacterial infections is increasing rapidly, limiting treatment options and often necessitating the use of next-line antimicrobials, including linezolid and daptomycin, against which resistance is also being reported [4,5]. Notably, MRSA has exhibited the most pronounced global increase in both AMR-associated and attributable mortality over the recent decades [1].

Considerable progress has been made in understanding the epidemiology, molecular mechanisms of resistance, and the activity of established and newer antimicrobial agents available for the management of Gram-positive infections. However, the continuous evolution of AMR, the emergence of resistance to newer antimicrobial agents, and the expanding range of therapeutic strategies have increased the complexity of clinical management. This review examines current therapeutic challenges in infections caused by three major Gram-positive pathogens—*Staphylococcus aureus*, *Enterococcus* spp., and *Streptococcus pneumoniae*—and discusses therapeutic strategies beyond vancomycin. Particular emphasis is placed on resistance to newer anti-Gram-positive agents, optimization of antimicrobial therapy, and emerging therapeutic approaches. To translate evolving evidence into clinical practice, the review provides a practical treatment algorithm for the management of resistant and difficult-to-treat Gram-positive infections and critically evaluates recent studies of combination therapy, including their potential role in overcoming resistance, enhancing antimicrobial activity, and improving clinical outcomes. Given the rapidly expanding armamentarium of anti-Gram-positive agents, the review focuses on selected antimicrobials for which resistance mechanisms and therapeutic strategies have been most extensively investigated (respective methodologies in Supplementary File S1).

## 2. Antimicrobial Resistance in Gram-Positives

### 2.1. Staphylococcus aureus

*S. aureus* constitutes a major public health concern, acting as both a commensal and an opportunistic pathogen causing infections in community and healthcare-associated settings. Approximately 30% of the general population is colonized on skin and nasal cavity, with certain populations including immunocompromised individuals, diabetic patients, the elderly, and people living with HIV, being at higher risk (up to 80%) of colonization [6]. It is responsible for a wide spectrum of infections ranging from skin and soft tissue infections to pneumonia, endocarditis and bloodstream infections with high mortality rates, with bacteremia alone showing a case fatality rate of 15–30% and causing approximately 300,000 deaths worldwide each year [7].

Following the introduction of methicillin in the early 1960s, MRSA emerged likely as a result of antibiotic selective pressure on pre-existing resistant strains [8]. Methicillin resistance occurs through the acquisition of staphylococcal cassette chromosome *mec* (SCC*mec*) elements via horizontal gene transfer, which carry a mec gene complex that alter penicillin-binding proteins (PBPs) which are essential for cell wall synthesis [9]. *MecA* gene encodes PBP2a which takes over the activities of endogenous PBPs and due to its low affinity for β-lactam antibiotics is a major contributor to MRSA resistance [10]. The prevalence and dissemination of MRSA have shifted over time, with new clones emerging across different countries and regions [11]. MRSA prevalence varies considerably across regions, with some Northern European countries reporting rates below 5%, reaching up to 25–50% in Southern and Eastern Europe and exceeding 70% in some East Asian regions [12,13,14]. MRSA has been a major contributor to difficult-to-treat infections, such as bacteremia, with its incidence rising in both healthcare-associated and community-associated settings. After a period of overall decline due to improvements in infection control, recent data suggest loss of previous progress [7,15]. Vancomycin is widely used to treat invasive MRSA infections; however, reliance on this agent led to the emergence of isolates with reduced susceptibility [16].

Vancomycin-resistant *S. aureus* (VRSA) and vancomycin-intermediate *S. aureus* (VISA) are defined as isolates with vancomycin minimum inhibitory concentration (MIC) of ≥16 μg/mL and 4 to 8 μg/mL, respectively, according to the Clinical and Laboratory Standards Institute (CLSI) breakpoints [17]. High-level vancomycin resistance is predominantly mediated by the *vanA* gene cluster, which encodes proteins responsible for the modification of the D-Ala-D-Ala terminal of the peptidoglycan precursor to D-Ala-D-Lac. This alteration results in a marked reduction in vancomycin binding, leading to loss of its bactericidal activity [18]. The *vanA* gene cluster is typically acquired via horizontal gene transfer from *Enterococcus* [19]. In contrast, VISA is associated with mutations that develop during vancomycin therapy, primarily affecting regulatory pathways of cell-wall biosynthesis and, in some cases, involving mutations in the RNA polymerase gene *rpoB* [20]. The prevalence of VRSA remains relatively low worldwide. However, a systematic review and meta-analysis demonstrated an increase over time, with the pooled prevalence rising from 1.2% before 2010 to 2.4% thereafter [21]. The same analysis reported the highest pooled prevalence of VRSA in the U.S. [21]. Reduced susceptibility phenotypes, including VISA and heterogeneous VISA (hVISA), are more frequently reported with pooled prevalences of 1.7% and 4.6%, respectively [21].

Therefore, vancomycin treatment failure is not solely attributable to antimicrobial resistance and this is particularly evident in hVISA infections. Treatment failure has been associated with higher vancomycin MICs, even within the susceptible range, as well as with the severity of infection and with intrinsic limitations of the drug, including poor tissue penetration, particularly in the lung and bone—and relatively slow bactericidal activity [22,23,24].

### 2.2. Enterococcus spp.

Enterococci are facultatively anaerobic Gram-positive cocci that typically exist as commensals in the gastrointestinal tract of humans. Due to their ability to develop resistance against multiple antibiotics, they have become a leading cause of nosocomial infections, particularly among immunocompromised individuals and patients in Intensive Care Units (ICUs) [25]. *Enterococcus faecalis* is the most frequently isolated species, accounting for 80–90% of nosocomial enterococcal infections, followed by *E. faecium*, which accounts for 10–15%, although its prevalence has been increasing in recent years [26,27]. These organisms are commonly associated with urinary tract infections, endocarditis, bacteremia, catheter-related infections, intra-abdominal and pelvic infections with increased mortality rates [28]. The emergence and spread of vancomycin-resistant enterococci (VREs) further complicate the management of these infections [28].

Vancomycin resistance is mediated by the acquisition of *van* gene clusters, which encode enzymes that alter the D-Ala-D-Ala moiety of peptidoglycan precursors to D-Ala-D-Lac or D-Ala-D-Ser depending on the acquired *van* gene cluster, thus reducing vancomycin binding affinity. The *vanA* and *vanB* gene clusters are the most clinically relevant, conferring high and variable levels of resistance. Additionally, enterococci exhibit intrinsic and acquired resistance mechanisms, including low-affinity PBPs and aminoglycoside-modifying enzymes [29,30]. Recent data from the European Antimicrobial Resistance Surveillance Network showed an increase in vancomycin-resistant *E. faecium* isolates from 10.4% in 2015 to 17.3% in 2018 [31].

VRE infections constitute a major public health concern, given their association with increased morbidity and mortality, limited therapeutic options, as well as their capacity for sustained transmission in healthcare settings, often resulting in prolonged outbreaks. Furthermore, VREs exhibit a notable ability to acquire and disseminate resistance determinants within and across species [25].

### 2.3. Streptococcus pneumoniae

*S. pneumoniae* is a leading cause of community-acquired pneumonia, especially among young children, the elderly and immunocompromised individuals. Beyond localized infections, it can also lead to severe invasive disease, including bacteremia and meningitis [32]. Overall, more than 2 million pneumococcal infections occur annually in the United States, resulting in more than 6000 deaths. More than 30% of these infections are caused by strains resistant to one or more clinically relevant antibiotics. In 2014, drug-resistant *S. pneumoniae* was estimated to account for approximately 900,000 infections and 3600 deaths [33]. Despite the availability of effective vaccines, macrolide-resistant *S. pneumoniae* along with macrolide-resistant Group A and penicillin-resistant Group B streptococci were included among WHO priority pathogens [2].

Widespread macrolide use is associated with increased macrolide resistance in *S. pneumoniae*, with reported prevalence varying geographically, ranging from below 10% to over 90% [34]. Additionally, the ability of *S. pneumoniae* to remodel its genome through the uptake and incorporation of exogenous DNA from other pneumococci or related oral streptococci has facilitated the dissemination of antibiotic resistance [35].

Macrolide resistance is primarily mediated by ribosomal target modification via *ermB*, which encodes a methyltransferase that alters the 23S rRNA and confers resistance to macrolides, lincosamides and streptogramin B, as well as high-level resistance to macrolides (MICs > 256 μg/mL) [36]. An additional mechanism involves active macrolide efflux mediated by the *mefE*/*mel* operon, resulting in lower-level resistance [37]. Less commonly, resistance occurs due to mutations in 23S rRNA or ribosomal proteins (L4 and L22) [38]. Dual macrolide resistance mediated by both ermB and mefE/mel is increasingly reported in *S. pneumoniae* worldwide [34].

Van-type vancomycin resistance has not been identified in clinical isolates of *S. pneumoniae* [37]. Although vancomycin resistance in *S. pneumoniae* is rare, the phenomenon of vancomycin tolerance has been observed in a few strains. This phenomenon was associated with defects in the autolytic pathway, preventing bacterial cell lysis despite inhibition of cell wall synthesis [39].

Table 1 summarizes mechanisms of resistance against commonly used antibiotics in Gram-positives.

## 3. Antimicrobial Strategies Against Gram-Positive

### 3.1. Vancomycin: The Last Frontier?

Vancomycin continues to be a key agent for the management of Gram-positive infections; however, it is associated with several limitations, including the increasing rates of reduced susceptibility and slow bactericidal activity. Its efficacy is further compromised in difficult-to-treat infections, particularly in biofilm-associated settings due to inadequate penetration [49]. High bacterial burdens impair vancomycin bactericidal activity, with pharmacodynamic studies showing that increasing inoculum sizes are associated with elevated vancomycin MICs and suboptimal Area Under Curve (AUC)/MIC ratios, thereby limiting therapeutic efficacy in infections characterized by large bacterial loads [50]. One major concern with vancomycin use is its narrow therapeutic window and the associated risk of nephrotoxicity. The reported incidence of vancomycin-associated acute kidney injury (AKI) varies across studies, ranging from 5 to 43%, while a meta-analysis demonstrated a relative risk of 2.45 (95% confidence interval, 1.69–3.55) for AKI among vancomycin-treated patients, with an attributable risk of 59% [51,52]. Notably, the trough concentrations of 15–20 mg/L previously recommended for severe MRSA infections have been associated with increased risk of nephrotoxicity [53]. Another concern is the phenomenon of vancomycin MIC creep, characterized by a gradual shift of *S. aureus* populations towards higher MICs despite remaining within the susceptible range [54]. Although the clinical significance of this phenomenon remains debatable, with large surveillance studies failing to demonstrate a consistent global increase in vancomycin MICs, several single-center studies have reported local shifts toward higher MICs [55,56]. A higher vancomycin MIC may compromise the achievement of the recommended pharmacokinetic/pharmacodynamic (PK/PD) targets and is associated with poor clinical outcomes [54,57]. These limitations may result in treatment failure and infection recurrence, leading to increased reliance on alternative antibiotics, for which resistance is also emerging.

### 3.2. Beyond Vancomycin

#### 3.2.1. Linezolid

Linezolid is the first synthetic oxazolidinone and exerts its antimicrobial activity by inhibiting bacterial protein synthesis, interfering with translation [58]. Linezolid binds to the peptidyl transferase center in the 50S ribosomal subunit, and alters the positioning of tRNA, interfering with its binding to the ribosomal A site, thereby hindering protein synthesis [58,59]. It demonstrates activity against Gram-positive pathogens, including MRSA, VRE, and Group A and Group B streptococci. It is approved for the treatment of VRE infections including bacteremia, as well as nosocomial pneumonia and severe community-acquired pneumonia (CAP) when methicillin-susceptible *S. aureus* (MSSA), MRSA or VRSA is suspected, and for both complicated and uncomplicated skin and soft tissue infections (SSTIs) [60,61,62,63]. Off-label uses include central nervous system infections, bone and joint infections and tuberculosis [64,65].

Linezolid resistance in Gram-positive bacteria occurs through chromosomal mutations and acquisition of transferable genes. A major mechanism involves mutations in domain V of the 23S rRNA, which result in reduced linezolid binding to the peptidyl transferase center. Additionally, mutations in ribosomal proteins L3, L4 and rarely L22, alter the ribosomal structure and interfere with antibiotic binding [66,67]. Moreover, resistance is often mediated by genes located on mobile genetic elements, thus facilitating horizontal gene transfer and resistance dissemination across bacterial species. Of importance, *cfr* gene encodes a methyltransferase that methylates adenine 2503 in 23S rRNA, and is associated with cross-resistance to multiple antibiotics, such as phenicols, lincosamides, oxazolidinones, pleuromutilins, and streptogramin A [68]. Another mechanism involves *optrA* and *poxtA* genes that encode ATP-binding cassette-F proteins which confer ribosomal protection by displacing the antibiotic from its binding site [69,70]. *Cfr* remains relatively uncommon among enterococci, although enterococci may serve as a reservoir for horizontal transfer to staphylococci. In contrast, *optrA* has emerged as the predominant transferable oxazolidinone resistance determinant among *E. faecalis*. *PoxtA* is more commonly associated with *E. faecium* and has been reported in clinical isolates across Europe, the United States, Pakistan and Turkey [66].

The reported resistance rates among Gram-positive pathogens remain generally low. The overall prevalence of linezolid resistance in *E. faecalis* and *E. faecium* was approximately 2.2% according to a meta-analysis [71]. In staphylococci, resistance occurs in 0.05% of *S. aureus* and 1.4% of coagulase-negative *Staphylococci* (CoNS), while reported rates for *S. pneumoniae* range from 0 to 4.9% [72,73]. Linezolid resistance appears to be associated with polymicrobial infections, prior exposure to glycopeptides and long and/or repeated courses of administration [74,75].

#### 3.2.2. Daptomycin

Daptomycin is a cyclic lipopeptide antibiotic that binds and inserts into the bacterial cell membrane in a calcium-dependent manner, resulting in membrane depolarization and subsequent loss of intracellular components [76]. This process leads to the intracellular inhibition of DNA, RNA, and protein synthesis, ultimately resulting in bacterial cell death [76,77]. Daptomycin may also interfere with cell division and cell wall synthesis [76]. It demonstrates a broad spectrum of activity against Gram-positive organisms including MRSA, glycopeptide-intermediate *S. aureus*, VRE and resistant streptococci [78]. Daptomycin is approved for the treatment of complicated SSTIs, infective endocarditis, and *S. aureus* bacteremia; however, it is also used off-label for the management of VRE infections, as well as bone and joint infections [79].

Intrinsic resistance to daptomycin has been described in some non-pathogenic organisms which contain enzymes that hydrolyze and inactivate daptomycin; however, this resistance mechanism has not been observed in clinically important bacteria where resistance is primarily acquired [77,80]. In *S. aureus*, resistance is primarily driven by mutations that increase the cell surface positive charge, leading to electrostatic repulsion of the daptomycin–calcium complex and impaired binding to the cell membrane. These mutational changes involve genes such as *mprF*, *yycG* (*walK*) and *rpoB/rpoC* [77]. Importantly, *mprF* mutations have been associated with cross-resistance to vancomycin [81]. In enterococci, daptomycin resistance is primarily driven by adaptive mutations affecting the LiaFSR cell envelope stress response pathway. Experimental disruption of LiaFSR signaling results in daptomycin hypersusceptibility and significantly delays the onset of daptomycin resistance, although resistance may eventually emerge through alternative mechanisms [45,82]. *E. faecalis* has developed a distinct resistance strategy associated with redistribution of cardiolipine microdomains away from daptomycin’s binding site, resulting in diversion of the drug from its target [80].

Despite emerging reports, resistance to daptomycin remains low, particularly among staphylococci where reported rates ranging from 0.1 to 0.3% [83]. The corresponding rate for enterococcal isolates is below 2%; however, the prevalence of resistance may be overestimated due to clonal dissemination of resistant strains in healthcare settings [77]. Prolonged treatment courses, high bacterial burden, inadequate treatment regimens, irregular drug supply and poor drug quality have been recognized as important risk factors for the development of daptomycin resistance [84]. Notably, resistance may occur in the absence of prior daptomycin exposure, particularly in isolates previously treated with vancomycin [77,84].

#### 3.2.3. Ceftaroline

Ceftaroline is a fifth-generation cephalosporin that, like other β-lactams, inhibits the bacterial cell wall synthesis by binding to the PBPs. It retains activity against MRSA, by binding allosterically to PBP2a through a side chain, inducing a conformational change that exposes the active site, thereby allowing inhibition [85]. Ceftaroline demonstrates activity against other multidrug-resistant Gram-positive pathogens, including hVISA, VISA, VRSA and *S. pneumoniae*, while exhibiting limited Gram-negative coverage [86,87,88]. The drug is approved by both the Food and Drug Administration (FDA) and European Medicines Agency (EMA) for the treatment of CAP and acute bacterial skin and skin structure infections (ABSSSIs) [89,90].

Resistance to ceftaroline remains uncommon, with data suggesting over 90% susceptibility among MRSA isolates [91,92]. However, sporadic reports highlight the emerging resistance, with studies conducted in Greece and India indicating higher resistance rates, potentially due to nosocomial spread of resistant MRSA strains [92,93,94,95]. Mutations in PBP2a can affect the activity of ceftaroline against MRSA [96], although recent evidence suggests that high-level resistance may arise through pathways triggered by prior carbapenem use, thereby involving rpoB mutations and alterations in other PBPs beyond PBP2a alone [97].

#### 3.2.4. Long-Acting Lipoglycopeptides (Dalbavancin/Oritavancin)

Dalbavancin shares a similar mechanism of action with other glycopeptides; however, its distinct structural and pharmacokinetic properties provide enhanced bactericidal activity, with 16–32-fold-greater potency than vancomycin and an increased half-life that enables a once-weekly dosing regimen [98,99]. It exhibits activity against a broad spectrum of Gram-positive pathogens, including staphylococci (MRSA and CoNS), streptococci, and vancomycin-sensitive enterococci. It has limited activity against *vanA*-type VRE; however, it retains activity against VRE carrying *vanB* gene cluster [45,100]. Dalbavancin is indicated for the treatment of patients with ABSSSI caused by Gram-positive pathogens [101].

Although dalbavancin resistance remains rare, recent evidence indicates that reduced susceptibility may develop during treatment, particularly in multidrug-resistant and biofilm-producing strains, as well as in invasive infections where higher MICs have been observed [102,103]. Additionally, the prolonged half-life of dalbavancin, that may result in sustained subinhibitory drug exposure toward the end of therapy, is likely to promote resistance development, facilitating the selection of less susceptible isolate subpopulations [104,105]. Resistance is primarily mediated by mutations in PBP genes, and regulatory systems such as WalKR system, where the disappearance of *walK* gene leads to increased cell wall thickness and reduced drug binding [102,104]. Notably, dalbavancin resistance is associated with cross-resistance to vancomycin and daptomycin, reflecting shared resistance pathways, as well as cross-susceptibility (see-saw effect) to various β-lactams [102,104,106].

Oritavancin is another semisynthetic lipoglycopeptide that is used for the treatment of ABSSSIs caused by susceptible Gram-positive bacteria, including MRSA, VISA and VRE [107]. Its antimicrobial activity occurs through multiple mechanisms, including the inhibition of transglycosylation and transpeptidation, as well as the disruption of the cell membrane [108].

To date, there is no evidence of resistance to oritavancin among clinical isolates, with oritavancin demonstrating potent and stable activity against Gram-positive pathogens (MIC_90_ range of 0.06 to 0.5 mg/L) over a 10-year period [109]. However, reduced susceptibility (MIC 8–16 μg/mL) has been observed in vitro among *Enterococcus* isolates exhibiting VanA and VanB phenotypes [107,110].

#### 3.2.5. Tigecycline

Tigecycline is a semisynthetic glycylcycline derived from minocycline, with an expanded spectrum of activity against Gram-positive, Gram-negative, anaerobic and atypical bacteria has been approved for the treatment of complicated SSTIs and IAIs, including MRSA and VRE infections. The drug‘s antibacterial activity is mediated through binding reversibly to the 30S ribosomal subunit, thereby preventing the incorporation of tRNA into the ribosomal A site and inhibiting bacterial protein synthesis [111,112,113].

In *S. aureus*, proposed mechanisms of tigecycline resistance include the overexpression of MepA efflux pump and mutations in *rpsJ* encoding the ribosomal S10 protein, with the latter also being associated with reduced tigecycline susceptibility in *E. faecium* [114,115]. Tigecycline resistance remains uncommon among MRSA isolates, with a pooled prevalence of approximately 0.4% according to a recent meta-analysis [116]. Isolated cases of tigecycline-resistant VRSA have been reported, as well as the recent emergence of tigecycline-resistant VRE, highlighting the need for ongoing resistance surveillance [117,118].

## 4. Strategies to Overcome Resistance

### 4.1. Optimize Vancomycin

Despite the challenges associated with vancomycin therapy, several strategies have been proposed in order to optimize treatment. Current consensus guidelines recommend AUC-guided monitoring with a target AUC/MIC of 400–600 mg·h/L for serious MRSA infections if MIC is ≤1 mg/L, as this range maximizes efficacy and minimizes toxicity [57,119]. This approach has replaced the previous trough-based approach, as previous studies have demonstrated that trough values may not accurately predict AUC values [57,120]. In order to achieve the recommended target, either first-order PK equations based on peak and trough concentrations or preferably Bayesian software programs can be employed [57]. Moreover, a loading dose of 25–30 mg/kg is recommended to optimize early attainment of target concentrations in selected cases, including critically ill patients, those admitted in ICUs, or patients receiving dialysis or renal replacement therapy; however, the initial dose must not exceed 3000 mg [57]. Also, continuous infusion may represent a useful alternative, especially among critically ill patients, as it may facilitate rapid attainment of target exposure and therapeutic drug monitoring [57]. Although continuous infusion exhibits pharmacokinetic advantages over intermittent dosing, comparative studies have not yet established a clear benefit in terms of toxicity and mortality [121,122,123].

### 4.2. Switch EARLY to Other Regimens

When vancomycin MIC exceeds 1 mg/L the probability of reaching the recommended AUC/MIC targets with conventional dosing becomes challenging, while dose escalation may increase the risk of toxicity [57]. Available data suggest that patients with MRSA bloodstream infection with vancomycin MIC ≥ 1.5 mg/L respond poorly to vancomycin treatment, and thus alternative options should be considered [124]. Current guidelines suggest that in cases where vancomycin MIC is >1 mg/L the decision to change therapy should be based on clinical judgment [57].

A recent meta-analysis showed that daptomycin treatment among patients infected with MRSA strains with MIC ≥ 1 mg/L was significantly associated with 40% lower odds of mortality compared to vancomycin treatment [125]. Furthermore, an early switch to daptomycin, within 3 days of vancomycin initiation, is associated with lower 30-day mortality, whereas delayed switching does not confer the same benefit [126,127]. Similarly, in cases of MRSA pneumonia with an inadequate response to vancomycin, switching to linezolid should be considered, given its demonstrated superiority over vancomycin in achieving clinical cure and microbiological eradication [128]. High-dose daptomycin is used as salvage and first-line therapy for complicated infections including endocarditis and bacteremia, primarily when caused by MRSA (when vancomycin MIC is >1 mg/L) and *Enterococcus* [129,130].

### 4.3. Combination Therapy

Given the emerging resistance to last-line antibiotics, combination therapy is increasingly employed for the management of difficult-to-treat infections. Beyond potential synergistic antibacterial effects, combination therapy has also been proposed as a strategy to prevent the emergence of resistance. This has been explored in persistent MRSA bacteremia where a second antibiotic is recommended on top of daptomycin in order to prevent daptomycin resistance from emerging, especially in cases with inadequate source control [131,132].

Combination therapy involving linezolid has been explored as salvage treatment for persistent Gram-positive infections; however, outcomes remain inconsistent, and clinical evidence is lacking with available data derived from case reports, small case series and preclinical studies [133,134]. Linezolid in combination with ertapenem exhibits high bactericidal and synergistic activity against MRSA [135]. In persistent MRSA bacteremia, when compared to a vancomycin-based combination regimen, linezolid-based salvage therapy demonstrated a higher success rate [136]. However, when used against VRE infections, linezolid combination therapy failed to demonstrate a survival benefit over monotherapy, but was associated with accelerated microbiological clearance and reduced hospital stay [137]. In a limited number of case reports, linezolid–daptomycin combination has been successfully employed as salvage therapy for MRSA infective endocarditis [138,139].

The addition of a β-lactam to daptomycin has been employed in selected MRSA cases, following the observed see-saw effect [140]. Compared with daptomycin monotherapy, combination with β-lactams was associated with improved clinical outcomes in high burden infections, including endocarditis and bone and joint infections [141]. Additionally, preliminary evidence suggests that daptomycin–ceftaroline combination therapy may improve mortality in selected patient populations; however, further studies are required to define the clinical characteristics that are associated with the optimal benefit [16,142]. As demonstrated in a case series study, ceftaroline–daptomycin combination may represent an effective option to accelerate clearance of refractory staphylococcal bacteremia [143]. Similar findings have been observed for the treatment of VRE infections with a daptomycin-and-β-lactam combination [144]. Moreover, combination therapy with daptomycin and fosfomycin has been associated with improved survival in patients with VRE bloodstream infection [145]. A limited number of case reports suggest that combination with rifampicin may also be effective against staphylococcal infections [146,147]. Daptomycin and cephalosporin (ceftriaxone, ceftaroline) combinations have been explored in experimental endocarditis models of streptococcal infective endocarditis with positive results [148,149].

Clinical data supporting the use of dalbavancin combination therapy are limited, with no clear advantage over monotherapy demonstrated to date. Comparable clinical outcomes have been observed between dalbavancin monotherapy and combination therapy suggesting limited additional benefit from combination therapy [150]. Combination regimens may be considered in selected difficult-to-treat infections including bone and prosthetic joint infections or subacute/chronic intravascular infections [151].

However, in many cases, combination therapy does not always lead to improved survival. In the CAMERA2 trial, the addition of antistaphylococcal β-lactam to standard of therapy despite reducing persistent bacteremia did not improve mortality and increased nephrotoxicity. Similarly, the addition of gentamicin did not demonstrate clinical benefit and its use is further limited by nephrotoxicity [152]. The ARREST trial found no clinical benefit with adjunctive rifampin in patients with *S. aureus* bacteremia [153]. Therefore, the optimal combination therapy remains to be defined. Table 2 summarizes findings of combination therapy studies against Gram positives.

### 4.4. Source Control

Effective management of Gram-positive infections also relies on adequate source control to reduce bacterial burden and improve treatment efficacy [102,154]. Early source control improves clinical outcomes and is associated with higher microbiological eradication and lower mortality [7,168]. Intravascular catheters and cardiac devices should be removed in case of persistent bacteremia, as their retention is associated with increased risk of hematogenous complications, while delayed removal may lead to bacteremia relapse [169,170,171,172]. Patients with prosthetic joints should receive specialist advice and management on a case-by-case basis, as the decision to remove the prosthetic material depends on the timing of bacteremia onset relative to surgery and various factors including comorbidities [172].

### 4.5. Prevent Resistance Emergence

Despite the introduction of several new agents in recent years, the antimicrobial pipeline remains insufficient for addressing the growing burden of antimicrobial resistance. This highlights the importance of introducing strategies that aim at preserving the activity of existing agents and prevent resistance emergence [4]. Antibiotic use, misuse and overuse remain the major drivers of antimicrobial resistance [173]. The implementation of antimicrobial stewardship programs that promote appropriate antibiotic selection, dosing and treatment duration is necessary [173]. Integrating rapid diagnostic testing with antimicrobial stewardship programs may provide a survival benefit, even in settings with well-established antimicrobial stewardship practices [174]. More importantly, rapid diagnostic testing and early pathogen identification may enhance antimicrobial de-escalation or in some cases provide the opportunity for watchful waiting, thereby limiting unnecessary antibiotic use [175,176].

### 4.6. Adjunctive Therapies

Beyond antimicrobial treatment, a number of adjunctive therapies are being investigated in order to improve clinical outcomes in difficult-to-treat Gram-positive infections. Given the association of biofilm formation with persistent infections and antimicrobial tolerance, several anti-biofilm strategies are currently under investigation [177,178]. These strategies focus on biofilm formation prevention through inhibition of bacterial adhesion and surface modification strategies, biofilm disruption, antimicrobial peptides, nanoparticle-based approaches and quorum-sensing inhibitors [177,178]. Moreover, bacteriophage therapy has emerged as a promising approach in the post-antibiotic era. Phages exhibit a narrow spectrum of activity with strong selectivity, specifically targeting and eliminating bacteria at the species or strain level, without disrupting patient’s normal microbiota [179,180]. A recent systematic review examining the efficacy and safety of phage therapy against Gram-positive infections identified encouraging clinical outcomes when phage cocktails or phage–antibiotic combinations were used against difficult-to-treat Gram-positive infections [180]. Phage therapy has shown promising activity against *S. aureus* infections including MRSA and VISA, with preclinical studies showing reduced bacterial burden and improved survival. However, further clinical studies are required to establish its benefits and role in clinical practice [181]. Last, host-directed therapies are being investigated as potential strategies to overcome antimicrobial resistance. This approach focuses on enhancing host immune responses and regulating inflammation [182].

## 5. Final Remarks

The management of Gram-positive infections with reduced vancomycin susceptibility remains challenging, reflecting a convergence of microbiological, pharmacological, and clinical limitations. Increasing reports of vancomycin MIC “creep” in *S. aureus*, even within the susceptible range, have been consistently associated with worse outcomes, including persistent bacteremia and higher mortality, thereby undermining the reliability of vancomycin as a first-line agent in severe infections [173,183]. In parallel, the emergence of hVISA/VISA phenotypes, alongside the global dissemination of VRE, highlights the adaptive capacity of Gram-positive pathogens under glycopeptide pressure [184]. From a PK/PD perspective, achieving optimal AUC/MIC targets early in the course of sepsis is frequently hampered by interpatient variability, augmented renal clearance, and toxicity constraints, particularly nephrotoxicity, which limits dose escalation and therapeutic optimization [57]. Furthermore, antimicrobial efficacy cannot be considered independently of source control, as persistent infection is often driven by retained infected devices, undrained collections, biofilm formation, or endovascular foci that may compromise treatment success despite appropriate antimicrobial exposure.

**Figure 1:**
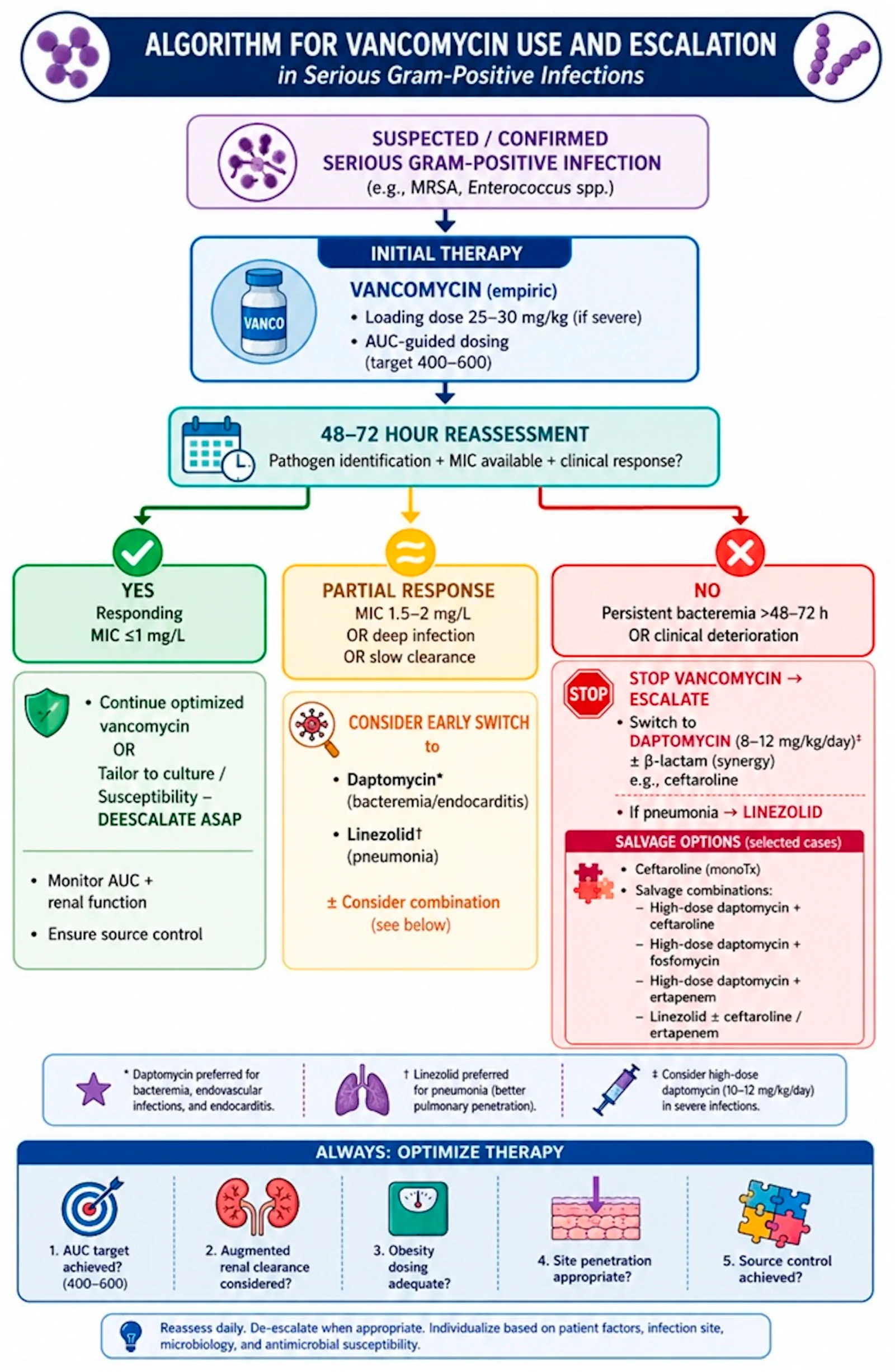
Algorithm for vancomycin use and escalation in serious Gram-positive infections.

These limitations have driven a paradigm shift toward earlier use of alternative agents such as daptomycin, linezolid, and ceftaroline, as well as combination strategies exploiting β-lactam synergy (“see-saw effect”), especially in persistent MRSA bacteremia and other high-inoculum infections [7,185] (Figure 1). Nevertheless, increasing reliance on these agents has also been accompanied by the emergence of reduced susceptibility to daptomycin and other last-line therapies, emphasizing that resistance evolution remains a dynamic and ongoing process. Although growing evidence supports combination approaches and individualized therapeutic escalation, robust comparative randomized data remain limited, and optimal sequencing, duration and composition of salvage regimens have yet to be standardized [186,187]. Overall, the available evidence remains heterogeneous, with some studies reporting no significant clinical benefit. Accordingly, therapeutic decisions should remain individualized, taking into account antimicrobial susceptibility, infection site, bacterial burden, source control, PK/PD considerations, and toxicity until higher-quality evidence becomes available.

Future perspectives lie in precision antimicrobial therapy integrating rapid molecular diagnostics, accelerated susceptibility testing, resistance genomics, real-time PK/PD modeling, and host-response biomarkers, to facilitate earlier and more individualized therapeutic decisions. In addition, novel agents, long-acting lipoglycopeptides, anti-biofilm approaches, bacteriophage-based therapies, and adjunctive immunomodulatory strategies may further expand the therapeutic armamentarium, although high-quality evidence is still needed to define their role in invasive and high-inoculum infections [188,189]. Important gaps remain, including limited activity against selected resistant pathogens, restricted indications for newer agents, and incomplete coverage of key organisms, particularly VRE, as highlighted in the latest WHO target product profiles for urgently needed antibiotics [188,190].

Ultimately, addressing antimicrobial resistance in Gram-positive pathogens requires a multifaceted and coordinated approach extending beyond the development of new drugs. Continuous resistance surveillance, implementation of robust antimicrobial stewardship programs, optimization of source control practices, and integration of One Health principles across human, animal, and environmental health sectors will be essential to preserve the effectiveness of existing therapies and improve outcomes in patients with severe Gram-positive infections.

## Figures and Tables

**Table 1:** Mechanisms of resistance to commonly used antibiotics in Gram-positives.

Antibiotic Class/Drug	Resistance Mechanism	Molecular Mechanism	Gene	Clinical Significance
***Staphylococcus aureus***				
β-Lactams [40,41]	β-lactamases	BlaZ a type A serine β-lactamase hydrolyzes the amide bond of the β-lactam ring in a two-step acylation–deacylation reaction cycle	*blaZ*	High hydrolytic activity against first generation penicillins
	PBP alteration	PBP2A substitutes the transpeptidase function of the four native PBPs which are highly sensitive to antibiotics	*mecA*	Methicillin resistance
Aminoglycosides [42]	Aminoglycoside modification	Inactivation of aminoglycoside antibiotics through the production of transferases AAC(6′)-Ie/APH(2″)-Ia, ANT(4′)-Ia, APH(3′)-IIIa	*aacA-aphD*, *aadD* and *aph*(*3′*)*-IIIa*	Most significant in clinical isolates: AAC(6′)-Ie/APH(2″)-Ia (resistance to gentamicin, tobramycin, amikacin)
Glycopeptides [4,43]	Cell wall modification	The terminal D-Ala-D-Ala in peptidoglycan precursors is converted to D-Ala-D-Lac, reducing antibiotic binding and preventing inhibition of cell wall synthesis	*vanA*	VRSA
	Cell wall remodeling	Alterations in regulatory pathways of cell-wall biosynthesis result in reduced vancomycin susceptibility	*walKR*, *rpoB*	VISA/hVISA
Tetracyclines [44]	Ribosomal protection	Tet(M) and Tet(S) proteins displace tetracycline molecules from the 30S ribosomal subunit	*tet*(*M*), *tet*(*S*)	Plasmid *tet(S)* is characteristic of VRSA strains
	Efflux pumps	Active removal of the antibiotic mediated by MFS transporters Tet(K), Tet(L), Tet(38), Tet(42), Tet(43), Tet(45), Tet(63)	*tet*(*K*), *tet*(*L*), *tet*(*38*), *tet*(*42*), *tet*(*43*), *tet*(*45*), *tet*(*63*)	Tet(K) spares minocycline
Fluoroquinolones [4,44]	Target site modification	Mutations to topoisomerase II and IV genes result in the synthesis of proteins with reduced fluoroquinolone binding	*gyrA*, *gyrB*, *parC*, *parE*	
	Efflux pumps	Active removal of the drug through transporters that are members of the MFS	*NorA*, *NorB*, *NorC* and *SdrM*	
Macrolides & lincosamides [44]	Target modification	Methylation of 23S rRNA prevents binding of the antibiotics to the 50S ribosomal subunit	*erm*(*A*), *erm*(*C*)	Inducible erm-mediated resistance may lead to clindamycin failure
	Efflux pumps	Active removal of macrolides through ATP-dependent efflux pumps	*msr*(*A*), *msr*(*B*)	
***Enterococcus* spp.**				
β-Lactams [29]	PBP alteration (or PBP4/5 mutations)	Expression of low-affinity PBPs (PBP4, PBP5) that bind weakly to β-lactams (accumulation of de novo point mutations in the penicillin-binding region of PBP4/5)	*pbp*	Intrinsic resistance (accumulated mutations in low-affinity PBPs contribute to acquired high-level penicillin resistance)
	β-lactamases	Identical to those of *S. aureus* encoded in β-lactamase transposon Tn*552*	*bla*	
Cephalosporins [29]	Two component regulatory systems	Resistance regulated via signaling systems affecting cell wall synthesis	*croRS*, *ireK*,*ireP* *ireB*, *murAA*	
Aminoglycosides [4]	Enzymatic modification	Acetylation/phosphorylation of the drug	*aph*(*2″*)*-Ic*, *aph*(*2″*)*-Id*, *aph*(*2″*)*-Ib*, *aph*(*2″*)*-Ie*	
	Ribosomal target modification	Methylation of 16S rRNA	*efmM*	Intrinsic resistance to tobramycin and kanamycin
Glycopeptides [29]	Cell wall target modification	Replacement of terminal D-Ala-D-Ala with D-Ala-D-Lac or D-Ala-D-Ser	*van* (*A*,*B*,*D*,*M*) or *van*(*C*,*E*,*G*,*L*,*N*)	*VanB* retains teicoplanin susceptibility
Macrolides, Lincosamides, streptogramins [45]	Ribosomal target modification	Methylation of 23S rRNA	*ermB*	Cross-resistance to macrolides, lincosamides and streptogramin B
Tetracyclines [4]	Efflux pumps	Active export of tetracyclines through membrane transporters	*tetK*, *tetL*	Spares tigecycline
	Ribosomal protection	Proteins homologous to bacterial elongation factors that displace tetracycline from its binding site	*tetM*, *tetO*, *tetS*	
Fluoroquinolones [46]	Target mutations	Point mutations in genes that encode the A subunits of DNA gyrase and topoisomerase IV	*gyrA*, *parC*	High-level fluoroquinolone resistance
	Efflux pumps	Active export of hydrophilic quinolones through NorA-like pump		
***Streptococcus pneumoniae***				
β-Lactams [47]	PBP alteration	Mosaic PBPs formed via interspecies homologous recombination reduce β-lactam binding affinity		PBPs 1A, 2X and 2B are associated with high-level penicillin resistance
Macrolides, lincosamides, streptogramins [4]	Ribosomal target modification	Methylation of 23S rRNA results in reduced antibiotic binding	*erm*	High-level macrolide resistance
	Efflux pumps	Active export of macrolides mediated by membrane transport system	*mefE*	Lower-level macrolide resistance
Fluoroquinolones [48]	Target modification	Chromosomal mutations in topoisomerase IV and DNA gyrase	*parC*, *gyrA*	
	Efflux pumps	Efflux mediated by MFS	*pmrA*	
Tetracyclines [4]	Ribosomal protection	Ribosomal protection proteins prevent tetracycline binding to the 30S subunit A- or P-site	*tetM*, *tetO*	

Abbreviations: PBPs: penicillin-binding proteins, MFS: Major Facilitator Superfamily, hVISA: heterogeneous vancomycin-intermediate *Staphylococcus aureus*, VISA: vancomycin-intermediate *Staphylococcus aureus*, VRSA: vancomycin-resistant *Staphylococcus aureus*.

**Table 2:** Studies of combination therapy against Gram-positives.

Study	Study Type/Size	Population	Combination	Outcome
**Persistent MRSA infection**				
Howden et al. (2004) [154]	Case series, 25 patients	Serious MRSA infections with reduced vancomycin susceptibility	Linezolid ± rifampicin ± fusidic acid	Glycopeptide failure in 76%. Linezolid-based regimens were effective
Jang et al. (2009) [136]	Retrospective study, 35 patients	Persistent MRSA bacteremia	Linezolid ± ertapenem salvage therapy vs. vancomycin + aminoglycosides or rifampicin salvage therapy	Linezolid-based salvage therapy had higher early microbiologic response and lower mortality rate
Park et al. (2012) [155]	Prospective study, 90 patients	Persistent MRSA bacteremia	Linezolid ± ertapenem salvage therapy vs. glycopeptide-based regimens	Bacteremia duration was longer for linezolid-based salvage therapy group. Linezolid-based salvage therapy demonstrated lower 30-day mortality rate
Dhand et al. (2011) [156]	Case series, 7 cases	Persistent MRSA bacteremia	Daptomycin + antistaphylococcal β-lactam	The addition of an antistaphylococcal β-lactam may be of benefit in refractory MRSA bacteremia
Sakoulas et al. (2014) [143]	Case series, 26 cases	Persistent/refractory staphylococcal bacteremia—mostly MRSA (includes 2 MSSA cases)	Ceftaroline + Daptomycin	Combination therapy accelerated bacteremia clearance
Claeys et al. (2015) [157]	Case series, 28 cases	MRSA infections—mostly persistent bacteremia	Daptomycin + TMP-SMX	After initiation of combination therapy, the median time to clearance of bacteremia was 2.5 days
Gritsenko et al. (2017) [158]	Case series, 5 patients	Persistent MRSA bacteremia	Vancomycin + Ceftaroline	Combination therapy may be considered when vancomycin monotherapy does not lead to microbiological and/or clinical improvement
Cortes-Penfield et al. (2018) [159]	Retrospective study, 17 patients	Persistent MRSA bacteremia	Daptomycin + Ceftaroline	Early combination therapy shortens prolonged bacteremia and may be helpful in securing favorable clinical outcomes
Hornak et al. (2019) [160]	Single-center, retrospective case series, 11 cases	Refractory MRSA bacteremia	Daptomycin + Ceftaroline or Vancomycin + Ceftaroline	Suggested combination as salvage therapy when other options fail
Patel et al. (2023) [161]	Retrospective, single-center cohort, 68 patients	Persistent MRSA bacteremia	Daptomycin + Ceftaroline vs. alternative therapy	Switching to daptomycin + ceftaroline after approximately 1 week of persistent bacteremia demonstrated similar efficacy to alternative therapy
Omori et al. (2024) [162]	Case series, 5 patients	Persistent MRSA bacteremia	Fosfomycin combination therapy	Fosfomycin combination therapy may be effective as salvage therapy for refractory MRSA bacteremia
Cabanilla et al. (2024) [163]	Retrospective cohort, 155 patients	High grade or persistent MRSA bacteremia	Vancomycin or Daptomycin monotherapy vs. Vancomycin or Daptomycin + Ceftaroline dual therapy	Dual therapy with ceftaroline did not reduce mortality rates or microbiological relapse
Althubyani et al. (2026) [164]	Retrospective cohort, 46 patients	Persistent MRSA bacteremia	Ceftaroline monotherapy vs. Ceftaroline + Daptomycin or Vancomycin	Combination therapy may reduce infection-related mortality
**VRE infections**				
Raad et al. (2001) [165]	Prospective study, 56 patients	VRE infections in immunocompromised patients	Quinupristin–dalfopristin + minocycline	Combination therapy is effective for VRE infections in cancer patients but it is associated with high frequency of arthralgia/myalgia
Chuang et al. (2018) [166]	Prospective observational study, 114 patients	VRE bacteremia	Daptomycin + β-lactam vs. daptomycin monotherapy	A high-dose combination therapy might improve survival
Irusta et al. (2020) [167]	Retrospective study, 85 patients	VRE bacteremia	Daptomycin + β-lactam vs. Linezolid or Daptomycin monotherapy	Did not find a significant difference in time-to-microbiological clearance
Chuang et al. (2022) [144]	Multicenter observational study, 430 patients	VRE (*E. faecium*) bacteremia	Daptomycin + β-lactam vs. Daptomycin monotherapy	Combination therapy was associated with a significantly higher rate of composite clinical success
Tseng et al. (2023) [145]	Retrospective study, 224 patients	VRE bacteremia	Daptomycin monotherapy vs. Daptomycin + Fosfomycin	Combination therapy improved survival rate
Siriwattanakowit et al. (2026) [137]	Retrospective study, 36 patients	VRE bacteremia	Linezolid-based combination therapy vs. monotherapy	Combination therapy did not demonstrate a survival benefit but was associated with accelerated microbiological clearance and reduced hospital stay

Abbreviations: MRSA: methicillin-resistant *Staphylococcus aureus*, MSSA: methicillin-susceptible *S. aureus*, VRE: vancomycin-resistant *Enterococcus*, TMP-SMX: trimethoprim/sulfamethoxazole.

## Data Availability

No new data were created or analyzed in this study. Data sharing is not applicable to this article.

## Supplementary Materials

The following supporting information can be downloaded at https://www.mdpi.com/article/10.3390/antibiotics15080780/s1, Supplementary File S1: Search methodology.

## Abbreviations

The following abbreviations are used in this manuscript:

AMR	Antimicrobial resistance
MDR	Multi-drug resistant
WHO	World Health Organization
MRSA	Methicillin-resistant *Staphylococcus aureus*
*S. aureus*	*Staphylococcus aureus*
HIV	Human immunodeficiency virus
PBPs	Penicillin-binding proteins
VRSA	Vancomycin-resistant *Staphylococcus aureus*
VISA	Vancomycin-intermediate *Staphylococcus aureus*
hVISA	Heterogeneous vancomycin-intermediate *Staphylococcus aureus*
CLSI	Clinical and Laboratory Standards Institute
MIC	Minimum inhibitory concentration
*E. faecalis*	*Enterococcus faecalis*
*E. faecium*	*Enterococcus faecium*
*S. pneumoniae*	*Streptococcus pneumoniae*
VRE	Vancomycin-resistant *Enterococcus*
ICU	Intensive care unit
CAP	Community-acquired pneumonia
AUC	Area under curve
PK/PD	Pharmacokinetic/pharmacodynamic
AKI	Acute kidney injury
MSSA	Methicillin-susceptible *S. aureus*
SSTIs	Skin and soft tissue infections
ABSSSIs	Acute bacterial skin and skin structure infections
IAI	Intra-abdominal infections
FDA	Food and Drug Administration
EMA	European Medicines Agency
TMP-SMX	Trimethoprim/sulfamethoxazole
CoNS	Coagulase-negative *staphylococci*
