# Supplementary material for: Antimicrobial Resistance in Major Gram-Positive Pathogens: Beyond the Vancomycin Border
Source: Antibiotics (Basel). 2026 Aug 13;15(8):780. doi: 10.3390/antibiotics15080780 (PMC13509171; doi:10.3390/antibiotics15080780)
Supplement: Supplementary file 1 [file antibiotics-15-00780-s001.zip › antibiotics-4443093-supplementary.pdf]

Supplementary File S1

Search Methodology

A literature search was conducted in PubMed until June 2026, in order to identify publications relevant to antimicrobial resistance in Gram-positive pathogens. The search strategy combined terms related to clinically relevant Gram-positive pathogens, including "*Staphylococcus aureus*", "MRSA", "*Enterococcus*", "VRE", "*Streptococcus*", antimicrobial resistance and resistance to last-line antibiotics, including "vancomycin resistance", "linezolid resistance", "daptomycin resistance", "ceftaroline resistance", "dalbavancin resistance", "oritavancin resistance", "tigecycline resistance", as well as therapeutic strategies, including "combination therapy", "salvage therapy". The reference lists of eligible manuscripts were also manually screened in order to identify additional relevant publications.

Publications were selected according to their relevance to the predefined scope of this review, with emphasis on epidemiology, resistance mechanisms, therapeutic optimization, combination therapy, and emerging therapeutic approaches in infections caused by *Staphylococcus aureus*, *Enterococcus* spp. and *Streptococcus pneumoniae*. Priority was given to landmark studies, systematic reviews, meta-analyses, randomized clinical trials, clinical guidelines and high-quality observational studies. Publications not written in English, studies involving pediatric populations, non-human studies and articles irrelevant to the objectives of the review were excluded.

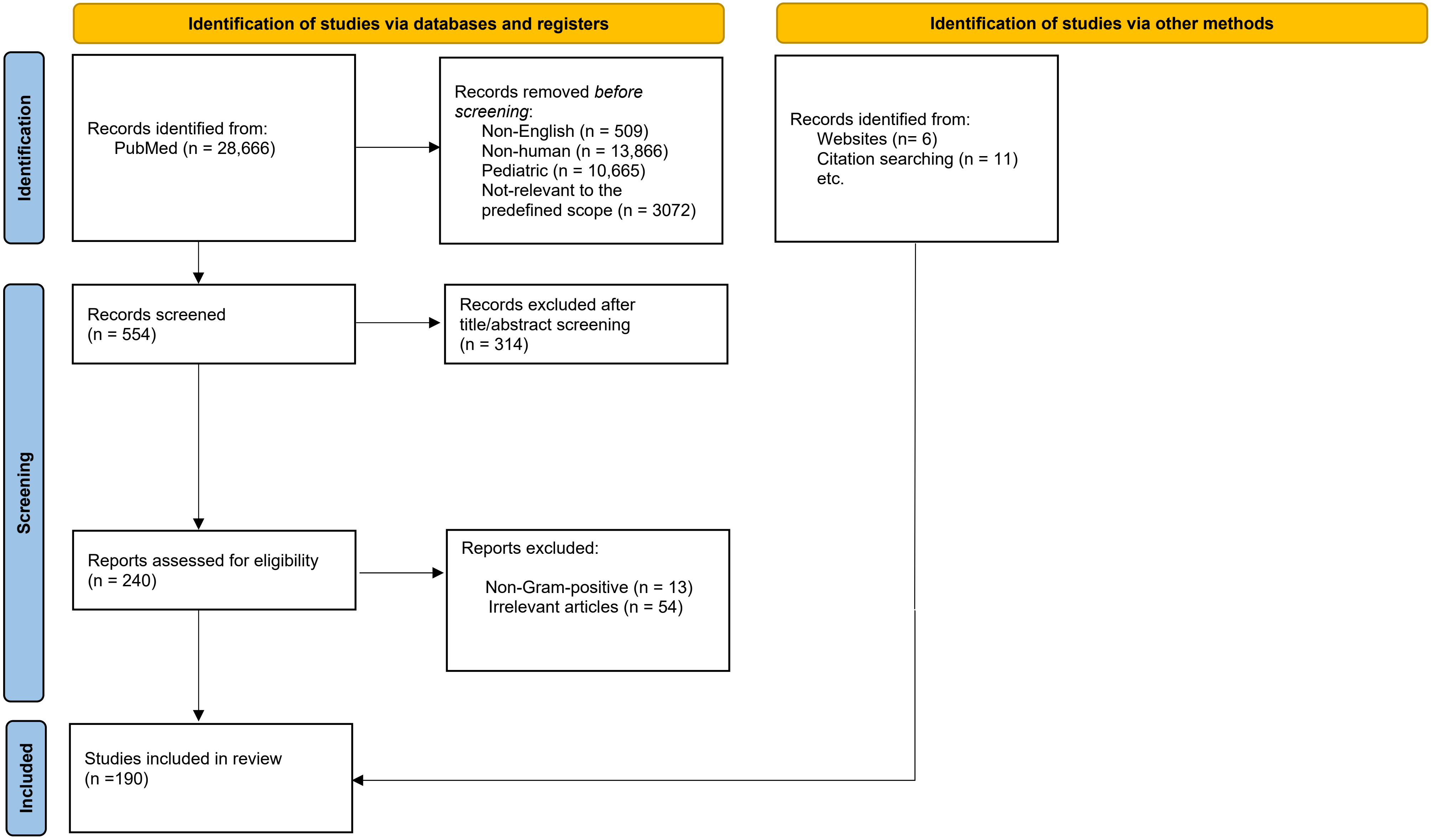
Identification of studies via databases and registers
Identification of studies via other methods
Identification
Records identified from:
PubMed (n = 28,666)
Records removed *before screening*:
Non-English (n = 509)
Non-human (n = 13,866)
Pediatric (n = 10,665)
Not-relevant to the predefined scope (n = 3072)
Records identified from:
Websites (n= 6)
Citation searching (n = 11)
etc.
Screening
Records screened
(n = 554)
Records excluded after title/abstract screening
(n = 314)
Reports assessed for eligibility
(n = 240)
Reports excluded:
Non-Gram-positive (n = 13)
Irrelevant articles (n = 54)
Included
Studies included in review
(n =190)
